# Dasatinib in the Management of Pediatric Patients With Philadelphia Chromosome-Positive Acute Lymphoblastic Leukemia

**DOI:** 10.3389/fonc.2021.632231

**Published:** 2021-03-25

**Authors:** Claudio Cerchione, Franco Locatelli, Giovanni Martinelli

**Affiliations:** ^1^ Hematology Unit, Istituto Scientifico Romagnolo per lo Studio e la Cura dei Tumori (IRST) IRCCS, Meldola, Italy; ^2^ Department of Pediatric Hematology/Oncology and Cell and Gene Therapy, IRCCS Bambino Gesù Children’s Hospital, Rome, Italy; ^3^ Department of Pediatrics, University of Rome, Sapienza, Rome, Italy

**Keywords:** acute lymphoblastic leukemia, Philadelphia positive, dasatinib, children, tyrosine kinase inhibitor

## Abstract

Acute leukemia is the most common cancer in childhood; in particular, acute lymphoblastic leukemia (ALL) represents roughly up to 80% of all cases of acute leukemias in children. Survival of children with ALL has dramatically improved over the last few decades, and is now over 90% (versus 40% of adult patients) in developed countries, except for in infants (i.e., children < 1 year), where no significant improvement was registered. Philadelphia positive ALL (Ph+ALL) accounts for around 3% of cases of childhood ALL, its incidence increasing with patient’s age. Before the era of tyrosine-kinase inhibitors (TKIs), pediatric Ph+ALL showed a worse prognosis in comparison to other forms of ALL, and was managed with intensive chemotherapy, followed, whenever possible, by allogenic hematopoietic stem cell transplantation (HSCT) in first morphological complete remission. TKIs have revolutionized the current clinical approach, which involves combinations of imatinib plus standard chemotherapy that can abrogate the negative prognostic impact conferred by the presence of BCR/ABL1 rearrangement, resulting in the probability of event-free survival (EFS) being significantly better than that recorded in the pre-TKI era. Long-term follow-up confirms these data, questioning the role of a real advantage offered by HSCT over intensive chemotherapy plus TKI in all Ph+ALL pediatric patients. Imatinib was the first generation TKI and the prototype of targeted therapy, but over the years second- (dasatinib, nilotinib, bosutinib) and third-generation (ponatinib) TKIs showed a capacity to overcome resistance to imatinib in Ph+ hematological neoplasms. Given the effectiveness of the first-in-class TKI, imatinib, also the second-generation TKI dasatinib was incorporated in the treatment regimens of Ph+ALL. In this manuscript, we will discuss the role of this drug in pediatric Ph+ALL, analyzing the available data published to date.

## Introduction

Through the application of reliable prognostic factors and risk-oriented treatment protocols, almost 85% of children with newly diagnosed acute lymphoblastic leukemia (ALL) can be cured today. The most common cause of treatment failure in pediatric ALL remains disease relapse, which occurs in approximately 15% of patients (1–8).

These improved patient outcomes in childhood ALL are due to many factors, including a better knowledge of the molecular lesions responsible for disease occurrence, monitoring of minimal residual disease (MRD), which represents a surrogate biomarker of leukemia cell sensitivity to chemotherapy, refined risk-adapted chemotherapy treatment and better results in patients given allogeneic hematopoietic stem cell transplantation (HSCT).

Despite these improvements in therapeutic management, ALL continues to impact the mortality rate of cancer in childhood. The outcome of refractory/relapsed ALL (r/rALL) remains, even nowadays, unsatisfactory and treatment must be diversified according to subsequent risk of treatment failure for children experiencing leukemia recurrence (8, 9). Innovative and flexible approaches need to be developed for timely treatment, along with more specific and effective drugs. However, at the same time, they need to be safely incorporated into the patient’s treatment. Such agents are likely to provide the best opportunities for improving the long-term survival and of the patient’s quality of life after ALL recurrence.

## Ph+ ALL

The discovery and definition of specific genetic abnormalities have increased knowledge of the biology of ALL. These have become the mainstay of clinical practice by providing relevant prognostic and predictive markers that influence treatment strategy and patient outcome. Although a wide range of genetic lesions have been discovered in childhood ALL, they are only partially relevant for prognosis (10). Response to treatment and prognosis of ALL can be strongly influenced by cytogenetic and molecular markers that can be associated with either good-risk or high-risk features. Among the cytogenetic/molecular abnormalities associated with a less favorable outcome, is the so-called Philadelphia chromosome, coming from t(9;22)(q34;q11), which is an encoded BCR-ABL1 fusion chimeric onco-protein with tyrosine kinase activity. The Philadelphia chromosome was first reported as the leading pathological alteration in chronic myeloid leukemia (CML). Then, in 1970, it was also found in ALL (11). 

Ph+ ALL is usually associated with poor prognosis in both adulthood and childhood (12). It accounts for 3–4% of pediatric ALL cases (almost exclusively of B-cell origin) and about 25% of adult ALL cases (13). The incidence of Ph+ALL increases with age, being higher in adolescents than in younger children (the BCR‐ABL1 fusion gene is detected in approximately 5-15% of adolescents). Before the era of TKIs, pediatric Ph+ ALL was associated with a dismal prognosis and was managed with intensive chemotherapy, followed, when possible, by HSCT in first remission (4). During recent decades, the availability of TKIs, in the context of CML, where the Philadelphia chromosome was first detected, had a dramatic impact on the management and prognosis of this disease (14). The revolutionary advancements in pharmacology provided by the advent of TKI led to the new concept of a targeted and “*personalized*” treatment of hematological neoplasms. ALL is not a unique disease, and its treatment strategy can be guided by the genetic and mutational landscape of the patient. The success of TKIs in CML has rapidly translated into attempts to treating other malignancies carrying the BCR-ABL1 fusion protein, including Ph+ ALL. Early published data have shown that in a Ph+ ALL pediatric population, imatinib combined with standard chemotherapy could reverse the negative impact on prognosis conferred by the presence of the BCR‐ABL1 fusion transcript, resulting in a significant improvement in the probability of event-free survival (EFS) (15, 16). Long-term follow-up confirmed these data, questioning the role of HSCT in first complete remission as compared to strategies based on the combination of intensive chemotherapy and TKI in this category of patients (17). Imatinib was the first generation TKI and remains the prototype of targeted therapy, but, over the years, second- (dasatinib, nilotinib, bosutinib) and third-generation (ponatinib) TKIs have shown a capacity to overcome resistance to imatinib in Ph+ hematological neoplasms (18). Given the effectiveness of the first-in-class TKI, namely imatinib, the second-generation TKI dasatinib was also incorporated into treatment regimens for Ph+ ALL.

## Targeting Protein Kinases

The human genome can encode for approximately 538 known protein kinases, whose activity maintains cellular function and cellular regulation through intracellular signaling pathways that are crucial for differentiation, survival, proliferation, metabolism, and cell-to-cell contact (19). Therefore, it is not surprising that protein kinases are one of the most relevant dysregulated molecules in human cancers, with several pathways that could lead to the proliferation of neoplastic cells in different types of hematologic and non-hematologic malignancies (19). Consequently, targeted therapy with small molecules and inhibitors against the activity of abnormal kinases is a leading method of treating hematological malignancies, and following imatinib, a first-generation TKI was approved for CML in 2001.

The Philadelphia chromosome results in the fusion gene BCR-ABL1 potentially existing in three principal isoforms. This is because it comes from different breakpoints on chromosome 22 in the BCR gene and encodes for three principal isoforms of aberrant protein kinases (namely, p190, p210, and p230) with distinct molecular mass (20). The frequency pattern of distribution of these isoforms is slightly different from CML to ALL and between adulthood and childhood (21, 22). In about 90% of cases of childhood Ph+ALL, t(9;22) mostly occurs in the *minor breakpoint cluster region* and produces a constitutional activate tyrosin kinase protein (of 190 kDa, p190 *BCR-ABL1*). The remaining cases are mainly represented by p210 isoforms (22).

Despite the different oncogenic activity in pre-clinical models between p190 and p210, there is no significant difference in terms of clinical outcome following chemotherapy in ALL patients harboring either of the two isoforms (23, 24). Regardless of the isoform, the chimeric BCR-ABL1 protein has direct effects on the oncogenic process by the ABL1 dysregulated and abnormal kinase activity that, in physiological conditions, is tightly controlled by a regulatory N-terminal region (25, 26). The chimeric BCR-ABL1 loses the regulatory region and, together with the boosting of BCR activity, physiological ABL1 functions are constitutionally activated. ABL1 is physiologically involved in a number of functions derived by interactions with other proteins. It is involved in the response of multiple extra and intracellular stimuli, playing a key role in cellular function, like cell-cycle or apoptosis (27). The final BCR-ABL1 mechanisms of transformation, as extensively studied in CML, are probably an altered cellular adhesion to stroma-cells and the matrix of bone marrow, triggering constitutively active mitogenic pathways together with inhibited apoptosis (28). As the majority of human neoplasms need multiple genetic steps to occur and determine the final neoplastic transformation, BCR-ABL1 is not the unique genetic alteration present in ALL and is not the unique neoplastic hit. However, given the effectiveness of TKIs in controlling the disease, BCR-ABL1 is potentially the major drive responsible for the abnormal proliferation of leukemia blasts in Ph+ALL. Therefore, targeting this dysregulated kinase activity represents a major treatment strategy for this leukemia.

Based on the mechanism of action, BCR-ABL1 kinase activity inhibition could be obtained through two major strategies: competitive inhibition and allosteric inhibition (29, 30). The first mechanism is provided by those ATP-competitive inhibitors, such as imatinib or dasatinib, whose binding site can be found in the catalytic cleft between the N-terminal lobe and C-terminal lobe kinase domain. These functional classes of molecules can be distinguished in type I and type II competitive inhibitors if they bind, respectively, to the activated/phosphorylated or inactivated/unphosphorylated conformation of kinase domain (31, 32). These inhibitors usually show scarce binding selectivity, which is particularly evident in type I over type II, providing inhibition or other kinases with consequently “off-target” side effects, like cardiac, pulmonary, gastrointestinal, and, especially in children, endocrine toxicity (33–36). The myristate binding domain or SH2-domain, are regulatory sites whose biological function is to quit an independent kinase activity, *via* different mechanisms (29). Therefore, the second type of inhibitor can bind to the regulatory sites that indirectly modulate the ATP-binding site conformation and activity in an allosteric fashion, providing highly selective kinase inhibition (37).

## Dasatinib Pharmacology

When discussing the pharmacological properties of a TKI it is relevant to compare it with the prototype of this class of compound, namely imatinib. Dasatinib is an oral TKI, whose inhibitor activity is also directed to other protein kinases (38). It differs from its precursor imatinib in several ways, involving a potency of inhibition BCR/ABL wild-type expressing cells *in vitro* greater than 300-fold, different activity profile on the non-BCR/ABL kinases targeted, and the presence of other specific anti-leukemic properties involving MAPK or BCL2 pathways (39). Regarding its higher inhibiting potency compared to imatinib, it is believed that this is associated with its ability to bind both activated and non-activated conformation of the BCL-ABL kinase, as a type II competitive inhibitor, compared to imatinib, whose target is the activated isoform only (38, 39). Acting as a multiple protein kinases inhibitor and not only a BCR-ABL-directed molecule, dasatinib can offer multiple pharmacodynamic antineoplastic effects. Therefore, an anti-leukemic action is also provided by blockage of the Stat-5 downstream pathway of BCR-ABL and SRC kinases family, which could reduce neoplastic proliferation and stimulate apoptosis (40). It could also interfere with p38 Map kinase of the MAPK family, which is demonstrated to be essential for the anti-leukemic effect of dasatinib (41).

*In vitro* data shows that dasatinib contributes to the anti-leukemic effect of imatinib-resistant neoplastic cells, even if some point mutations in BCR/ABL confer several degrees of resistance to dasatinib, with the maximum resistance displayed by T315I mutation, as also to the majority of available TKIs (42, 43). Off-target effects involving other kinases and targets are recognized as being responsible for some adverse events (AE) of dasatinib administration. The activity of hematopoietic cells is affected by the direct interaction of dasatinib with the BTK and TEC kinases, resulting in an impaired B- and T-cell development effect (44, 45). Pleural effusion, with characteristic lymphocyte-rich fluid, is a relatively common (20-35% of patients) AE reported with dasatinib treatment and is also probably caused by a specific immune-mediated off-target pharmacodynamic effect involving the PDGFR-beta pathway (46, 47).

Dasatinib has been shown to penetrate the central nervous system (CNS) at considerably higher levels, as confirmed also by more recent studies (48, 49).

## Dasatinib in Pediatric Ph + ALL—Clinical Experiences

### Phase I Trial

Zwaan et al. conducted a phase I trial in pediatric patients affected by imatinib-resistant or intolerant Ph+ CML, relapsed and refractory Ph+ ALL and relapsed Ph+ AML (50), in which dasatinib was administered in once-daily dose-escalation (starting from 60 until 120 mg/m2).

The efficacy and safety were comparable to adult results, with no response in Ph-negative relapsed/refractory ALL or AML. 60 mg/m^2 and 80 mg/m^2 once-daily were selected for phase II trials in Ph+ ALL.

### Phase II Trial—COG AALL0622

Slayton et al. conducted a phase II trial (COG AALL0622) in newly diagnosed children with Ph+ ALL, with dasatinib replacing imatinib on day 15 of induction. It was administered in combination with the same chemotherapy approach used in COG AALL0031 (51). Endpoints were safety and feasibility in 1-30-year-old patients. HSCT was recommended in high-risk/slow responder patients, and also in patients with a matched-family-donor independent from response. Standard-risk patients lacking an HLA-matched donor were managed with a combination of chemotherapy and dasatinib for 120 additional weeks, while CNS positive patients underwent cranial irradiation. Dasatinib plus chemotherapy showed good tolerability and outcomes similar to imatinib in COG AALL0031 (5-year OS 86% ± 5% overall, 87% ± 5% for high-risk patients); 5-year rate (± SD) of CNS relapse was 15% ± 6%. These findings confirm data obtained using imatinib and chemotherapy, with the recommendation to reserve HSCT only to slow responders, suggesting IKZF1 as a new biomarker whose potential role should be further investigated.

### COG AALL1131

The COG Trial (AALL1131) explored the role of dasatinib in newly diagnosed, high-risk Ph-like B-ALL, harboring ABL-class lesions (52). The authors identified new rearrangement partners which could be potential targets. This needs to be better and further explored in new trials aimed at detecting specific alterations in this particular subset.

### Phase II Trial—CA180-372

CA180-372 was an international phase 2 clinical trial, aiming to explore the combination of continuous daily dasatinib (daily dose of 60 mg/m^2 from day 15 of induction) plus EsPhALL chemotherapy in pediatric Ph+ ALL (53). Minimal residual disease (MRD) was evaluated at day 78, at the end of phase 1b, by several methods (Ig/TCR PCR, flow cytometry, and BCR - ABL1 RT-PCR). Patients who remained MRD positive at any detectable level after three additional high-risk chemotherapy blocks were candidates to receive HSCT in first complete remission (CR1), while dasatinib maintenance was optional. The other patients received a combination of dasatinib plus chemotherapy for 2 years, with cranial irradiation limited to CNS3 patients. This combination was safe and effective (in terms of 3-year EFS) in pediatric Ph+ ALL patients, with 14% who underwent HSCT in CR1, versus 80% in the EsPhALL trial.

### Total XVI Study

Jeha et al. designed the Total XVI Study (54, 55), comparing the outcome of Ph+ ALL in the pre-TKIs era versus TKIs-based treatments. TKIs (including dasatinib) were administered, starting from day +22 of induction therapy during all treatment phases, showing significant results in terms of MRD if compared with chemotherapy alone, in terms of 5-year EFS (68.6 ± 19.2% and 31.6 ± 9.9%, respectively (P = .022), confirming that the administration of TKIs in the early phases of treatment improves the outcome of pediatric Ph+ ALL.

## Dasatinib Versus Imatinib in the Treatment of Pediatric Philadelphia Chromosome–Positive Acute Lymphoblastic Leukemia—A Randomized Clinical Trial

Shen et al. (49), designed an open-label, phase 3, randomized clinical trial, including 225 patients from 20 hospitals in China. The trial examined whether dasatinib, at a daily dosage of 80 mg/m2, is more effective than the first-generation inhibitor imatinib mesylate, at a daily dosage of 300 mg/m2. It aimed to improve event-free survival in children with Philadelphia chromosome–positive ALL who had received intensive chemotherapy without prophylactic cranial irradiation (the secondary outcomes were relapse, death due to toxic effects, and overall survival).

The 4-year event-free survival and overall survival rates were 71.0% (95% CI, 56.2%-89.6%) and 88.4% (95% CI, 81.3%-96.1%), respectively, in the dasatinib group and 48.9% (95% CI, 32.0%-74.5%; P = .005, log-rank test) and 69.2% (95% CI, 55.6%-86.2%; P = .04, log-rank test), respectively, in the imatinib group. The 4-year cumulative risk of any relapse was 19.8% (95% CI, 4.2%-35.4%) in the dasatinib group and 34.4% (95% CI, 15.6%-53.2%) in the imatinib group (P = .01, Gray test), whereas the 4-year cumulative risk of an isolated central nervous system relapse was 2.7% (95% CI, 0.0%-8.1%), excellent control of central nervous system leukemia without the use of prophylactic cranial irradiation, in the dasatinib group and 8.4% (95% CI, 1.2%-15.6%) in the imatinib group (P = .06, Gray test). There were no significant differences in the frequency of severe toxic effects between the 2 treatment groups.

To date, this is the first clinical trial comparing the use of dasatinib versus imatinib in pediatric Ph-positive ALL settings, encouraging a switch in future studies.

## Consensus Paper

In a consensus paper, major experts in Ph+ ALL agree that HSCT remains the standard of care in adult patients. It outlined improved outcomes, thanks to the use of TKIs in frontline therapy, but with many patients still relapsing after the allograft. TKIs-based maintenance post-HSCT can reduce relapse risk and should be considered a valuable option (56). Future studies addressing the same issue in a pediatric population are needed, although some clinical experiences support the positive role of the drug in this clinical setting (57).

## Dasatinib as a Bridge to the Second Allograft in Post HSCT Relapsed Ph+ALL

A case report showed a Ph+ALL patient with early relapse after first HSCT, who was given dasatinib single agent treatment, achieving complete molecular remission, which persisted for 12 months after the second HSCT, with acceptable tolerability (58).

Another clinical case explored dasatinib in early relapsed Ph + ALL post HSCT. In this patient, after complete molecular response, dasatinib was used as a bridge to a second successful transplant, also showing a very good safety profile (59).

## Novel Combinations: The Next Future of Dasatinib-Based Treatment

A novel approach could be the combination of dasatinib plus ABT-199/venetoclax, which is a BCL2 (protein B-cell lymphoma 2) inhibitor. It showed improved antileukemic efficacy with equivalent tolerability if compared to either of the single agents in Ph+ ALL xenografted immunodeficient mice (60). This combination showed high synergism *in vitro*, with the decrease of cell viability and the induction of apoptosis in Ph + ALL, and, thanks to multikinase inhibition, it was shown to add the advantage of inducing Lck/Yes novel tyrosine kinase (LYN)-mediated proapoptotic BCL-2-like protein 11 (BIM) expression and inhibiting up-regulation of antiapoptotic myeloid cell leukemia 1 (MCL-1), potentially overcoming venetoclax resistance. These data are encouraging and clinical trials exploring this interesting combination are planned for the future. 

## Conclusions

In recent years there has been important progress in the management of pediatric ALL, thanks to risk-adapted protocols and CNS prophylaxis, while the prognosis for those with Ph+ALL remained unfavorable until the beginning of the TKIs era.

The second-generation TKI dasatinib, an oral inhibitor of chimeric BCR-ABL oncogenic kinase with multi-inhibitor activity, showed improved outcomes if used in combination with the standard chemotherapy approach. Moreover, in a post-HSCT setting, it could have potential benefits in the maintenance of this condition, but more solid data and further studies are required.

Preliminary data about the combination with other molecules, such as the BCL2-inhibitor venetoclax, are promising and confirm the need for further exploration of these combinations, which could form the backbone of new risk-adapted/MRD-driven clinical trials in relapsed Ph+ALL.

## Author Contributions

All authors have made substantial contributions to: the conception and design of the study, or acquisition of data, or analysis and interpretation of data; drafting the article or revising it critically for important intellectual content; and approved the final submitted version.

## Conflict of Interest

The authors declare that the research was conducted in the absence of any commercial or financial relationships that could be construed as a potential conflict of interest.
